# Maintenance therapy after first‐line therapy for ovarian cancer: Quantitative effectiveness

**DOI:** 10.3322/caac.70057

**Published:** 2025-12-09

**Authors:** Susana M. Campos, Jessica DiSilvestro, Stephanie V. Blank

**Affiliations:** ^1^ Gynecology Oncology, Department of Obstetrics and Gynecology Dana‐Farber Cancer Institute Boston Massachusetts USA; ^2^ Gynecology Oncology, Department of Obstetrics and Gynecology Tufts Medical Center Boston Massachusetts USA; ^3^ Gynecology Oncology, Department of Obstetrics Gynecology, and Reproductive Science, Icahn School of Medicine at Mount Sinai New York New York USA

For patients with ovarian, peritoneal, and fallopian tube carcinoma, completing first‐line treatment, maintenance therapy aims to stave off the risk of recurrence with the hope it will improve overall survival. Multiple trials, including the Gynecologic Oncology Group GOG‐0218 trial,^1^, ^2^ which investigated the vascular endothelial growth factor inhibitor (VEGFi) bevacizumab; SOLO1^3^, ^4^, ^5^ (olaparib; ClinicalTrials.gov identifier NCT01844986); PRIMA^6^, ^7^ (niraparib; (ClinicalTrials.gov identifier NCT02655016); and PAOLA‐1^8^ (bevacizumab plus olaparib (ClinicalTrials.gov identifier NCT24777644) have uniquely and independently played pivotal roles in shaping the role of maintenance therapy in this patient population.

Specifically, PAOLA‐1^8^ investigated whether there was value in a combined maintenance strategy. This trial randomly assigned people with ovarian cancer (both homologous recombination‐proficient [HRP] and homologous recombination‐deficient [HRD]) to receive either maintenance bevacizumab plus the poly (ADP‐ribose polymerase) inhibitor (PARPi) olaparib or bevacizumab plus placebo. Compared with bevacizumab plus placebo, there was a significant benefit in progression‐free survival (PFS) among patients with HRD disease who received bevacizumab plus olaparib (hazard ratio [HR], 0.41; 95% confidence interval [CI], 0.32–0.54). Among those with HRD disease because of a *BRCA1* or *BRCA2*  mutation, combined treatment resulted in an overall survival benefit (HR, 0.60; 95% CI, 0.39–0.93); this was not reported among those who had HRP disease (HR, 1.19; 95% CI, 0.88–1.63). Despite these results, notable limitations included the omission of a third arm in which participants received a PARPi alone and, as such, questions regarding the true as well as the *quantitative effectiveness* of bevacizumab plus olaparib versus treatment with olaparib alone remained unanswered. The only data to suggest a possible benefit to the combination come from an indirect comparison^9^ of SOLO‐1 and PAOLA‐1, and the study suggested that adding bevacizumab to olaparib was associated with a *numerical*, although not statistically significant, improvement in PFS (HR, 0.71; 95% CI, 0.45–1.09).

In this issue of *CA: A Cancer Journal for Clinicians*, we get the results of FZOCUS‐1, a randomized trial testing a PARPi, fuzuloparib, with a VEGFi, apatinib, as maintenance treatment after first‐line therapy in ovarian cancer.^10^ Participants were stratified by the presence or absence of germline *BRCA1* or *BRCA2* mutation and by response to primary treatment. They were randomly assigned 2:2:1 to one of three maintenance arms: fuzuloparib plus apatanib, fuzuloparib plus placebo, or placebo plus placebo.

Compared with placebo, treatment with either fuzuloparib plus placebo (HR, 0.58; 95% CI, 0.44–0.75) or fuzuloparib in combination with apatinib (HR, 0.57; 95% CI, 0.44–0.75) improved PFS. Although the addition of apatinib to fuzuloparib did not prolong PFS relative to fuzuloparib alone with statistical significance in any group, the lack of effect was most apparent in the non‐HRP groups. For example, the HR for patients who had pathogenic *BRCA* variants was 1.11 (95% CI, 0.69–1.79), 1.04 (95% CI, 0.83–1.32) for the overall population, and 1.09 (95% CI, 0.83–1.44), for those who had HRD disease. In the HRP group, a *nonstatistically significant* improvement in PFS of 11 months with combination therapy versus 16.6 months with dual therapy (HR, 0.73; 95% CI, 0.45–1.19) suggested *a PFS trend in favor of the combination therapy*, according to the authors.

Although this trial could appear to settle the question of whether there is a benefit to combining a PARPI and a VEGFi as maintenance treatment, a further critical look is necessary. First, the authors opted to use apatinib rather than the more widely used VEGFi bevacizumab. Although they assumed they were equivalent in both efficacy and toxicity, they have not been directly compared in the context of ovarian cancer. In addition, the authors highlight that an early phase study had reported a drug–drug interaction between fuzuloparib and apatinib: when used in combination, the exposure of apatinib was reduced by 48%–65% compared with its use as a single agent. This prompted a lower starting dose of fuzuloparib. In doing so, the dose of fuzuloparib used in the combination arm was 33% lower than what was used as a single agent, and this necessary attenuation of the PARPi may have contributed to the lack of a greater benefit for the combination. Suggestions that this might be relevant come from the earlier studies of olaparib, in which dosing at 100 mg orally twice a day was met with inferior results relative to dosing at 400 mg twice a day.

Ultimately, it is unclear whether the result of this trial brings us nearer to resolving the unanswered question of whether combination PARPi plus VEGFi treatment is better than single‐agent PARPi maintenance treatment. Therefore, we look forward to other frontline maintenance trials enrolling that aim to address this question, including Nirvana (niraparib vs. niraparib plus bevacizumab [ClinicalTrials.gov identifier NCT05183984]; AGO‐OVAR 28 (niraparib vs. niraparib plus bevacizumab [ClinicalTrials.gov identifier NCT05009082]); and MITO 25 (bevacizumab vs. bevacizumab plus rucaparib vs. rucaparib [ClinicalTrials.gov identifier NCT03462212]). This question will only be answered with statistical, and not *numeric*, significance or trends toward significance because the assumption should be that the benefit of adding the second drug needs to be proven.

## CONFLICT OF INTEREST STATEMENT

The authors declare no conflicts of interest.
